# A novel soft-tissue *in vitro *model for bisphosphonate-associated osteonecrosis

**DOI:** 10.1186/1755-1536-3-6

**Published:** 2010-04-01

**Authors:** MA Scheper, R Chaisuparat, KJ Cullen, TF Meiller

**Affiliations:** 1 Department of Oncology and Diagnostic Sciences and the Marlene and Stewart Greenebaum Cancer Center, University of Maryland Medical Center, 650 W Baltimore St, Baltimore, MD, 21201, USA; 2 Department of Oral Pathology, Chulalongkorn University, 254 Phyathai Rd, Patumwan, Bangkok, 10330, Thailand; 3 Marlene and Stewart Greenebaum Cancer Center, University of Maryland Medical Center, 22 S Greene St, Baltimore, MD, 21201, USA

## Abstract

**Background:**

Bisphosphonate (BP)-associated osteonecrosis of the jaw (ONJ) has been reported in patients receiving intravenous BP, particularly zoledronic acid (ZA). The purpose of this study was to develop an *in vitro *model representative of the effects BP has on soft tissue secondary to its release from bone. Human gingival fibroblasts and oral epithelial cell lines were exposed to various concentrations (0-10 μM) of ZA using dentine discs (DDs) as a direct carrier of BP, which were exposed for 24 hours to ZA in normal medium (NM), washed in phosphate-buffered saline (PBS) and placed in a new co-culture with the cells. The cells were allowed to proliferate until they grew over the bone discs and then the discs either were left unchelated, or were chelated using 0.001% EDTA or EGTA to release BP from the discs and to observe the cellular effects. Direct effects were determined using direct and fluorescent imaging. Apoptotic effects were determined by vital stain, terminal dUTP nick-end labeling, and annexin V studies. The effect on cell proliferation was determined by mitochondrial tetrazolium salt assay. The level of BP release was determined based on the effect of BP directly on cells, using the DDs or the supernatant fluids resulting from chelation.

**Results:**

A dose-response effect was seen on imaging, and effects on apoptosis and cell proliferation were observed with increasing ZA concentrations liberated from the DDs, particularly after calcium cleavage and release of ZA from the DDs with a variety of chelating agents. Apoptotic effects were observed microscopically after chelation at 24 hours. Release of ZA was confirmed by extracting medium from non-chelated and chelated cell culture models with DDs and applying this medium to untreated fresh cell cultures, providing appropriate controls.

**Conclusions:**

The results from this study demonstrate that low concentrations of ZA released from bone can rapidly and directly affect the oral mucosal tissues, initially through the induction of apoptosis and long term through the inhibition of cell proliferation. These findings provide an *in vitro *model for a soft-tissue mechanistic component in the initiation and/or progression of ONJ.

## Background

Bisphosphonates (BP) are synthetic analogues of the naturally occurring pyrophosphate, which are able to chelate calcium ions [1-3]. Zoledronic acid (ZA), a third-generation, nitrogen-containing BP, is one of the most potent available [4,5]. Its high affinity for calcium crystals allows this pyrophosphate to bind hydroxyapatite of bone and inhibit osteoclast-mediated bone resorption, a property that has provided the rationale for their use as skeletal protectors of cancer-mediated, cytokine-induced hypercalcemia [1-3,6,7]. In addition to malignancies such as multiple myeloma [8] and solid tumors at various stages [3], other specific conditions such as osteoporosis [4] and Paget's disease have been the focus for BP therapy.

One of the more recently reported serious adverse effects of ZA treatment is osteonecrosis of the jaw (ONJ), a condition first recognized in 2003 as a complication associated with BP therapy [9] and originally thought to involve the anti-angiogenic effects of ZA [10-12]. The reported incidence of ONJ ranges from 1.3% to 19%, with a higher frequency in the mandible than in the maxilla [13-21]. ONJ occurs in a dose- and time-dependent manner, with cases being more prevalent in patients receiving intravenous (IV) dosing and for longer periods of (10-59 months) [13-18]. Although ZA is known to preferentially deposit in areas of bone formation, the pathophysiologic mechanisms of ONJ are not fully elucidated [22]. One hypothesis suggested that the microenvironment surrounding active osteoclasts is strongly acidic, inducing the release of the BP from the bone surface and creating high local BP concentrations [2].

After administration of a single IV dose (2-16 mg) of ZA, most of the drug is plasma-bound, with 40% of the dose recovered in the urine within 24 hours, provided there is normal renal function. The plasma half-life ranges from 0 to 87 hours, with terminal elimination lasting up to 146 hours. The remainder of the dose is presumed to be bound to the bone, where it is slowly released over time, resulting in low plasma levels [23,24]. Currently, the oral mucosal levels of ZA are unknown; however, various studies suggest a range of 1-10 μM/L peak plasma concentrations after a 15 minute IV infusion, with higher concentrations expected in the bony microenvironment [25-27]. Recently, we developed a novel bioassay measuring the levels of BP released from discarded ONJ bone, using chelation with EDTA and EGTA as the mechanism for release [28]. In this study, we measured local levels of BP corresponding to approximately 0.4 to 4.5 μM in bone in patients taking BP [28]. These synthetic calcium chelators result in free BP in the supernatant fluids, which is capable of inducing apoptosis and suppressing cell proliferation compared with controls. The chelating agents (EDTA and EGTA) were selected to mimic the effects that saliva may have on the release of BP from bone, so that we could prove that when bound to bone or calcium, BP is harmless, but when released by competitive chelation, the free BP acts directly on the mucosal cells. This has been proven by our previous work and confirmed recently [29]. Importantly, our model has appropriate controls for the use of EDTA and EGTA.

In approximately 60% of cases of ONJ, there is a reported previous dental procedure or trauma; however, up to 40% of patients with ONJ have no such history. In fact, many of the cases of ONJ occur at sites away from teeth on otherwise normal undisturbed tissues [13,30]. The fact that ONJ occurs as both a wound-healing phenomenon and a spontaneous occurrence supports our suggestion that the pathogenesis is probably multifactorial.

Reid *et al*. [31] initially suggested the possibility of soft-tissue toxicity from BP in a 2007 editorial. This concept conforms to the clinical condition most commonly known as ONJ, which is observed as a mucosal dehiscence leading to the formation of a superficial mucosal ulcer, which progresses and results in detectable bone exposure. The ulcerated area continues to extend with time, leading to bone necrosis and sequestration [14]. We hypothesize that this effect occurs through the localized release of free BP from the bone surface, raising the concentration of BP in the interface microenvironment between bone, periosteal cells and the oral mucosa. This results in the apoptosis of mucosal cells and exposure of bone to the oral microbial environment, resulting in a mild to moderate surface osteomyelitis and prolonged ONJ.

Our model provides an *in vitro *mechanism for studying the initial effect of ZA release from bone on cells of the oral mucosa; that is, the highly specialized cell lines derived from oral epithelial and gingival fibroblast cells. We selected concentrations of ZA that are representative of the clinical plasma levels predicted after any IV infusion to validate our model and confirm our hypothesis that the direct effects of low levels of ZA released from bone induce mucosal cell apoptosis and inhibit cellular proliferation. This model may constitute an initiating mechanism for ONJ. In this study, we assessed the apoptotic and proliferative effects of ZA release from our bone model on oral mucosal cells.

## Results

### ZA-induced apoptosis and cell-growth inhibition

#### Initial analysis of apoptosis, fluorescent confirmation with rhodamine

**Figure 1:**
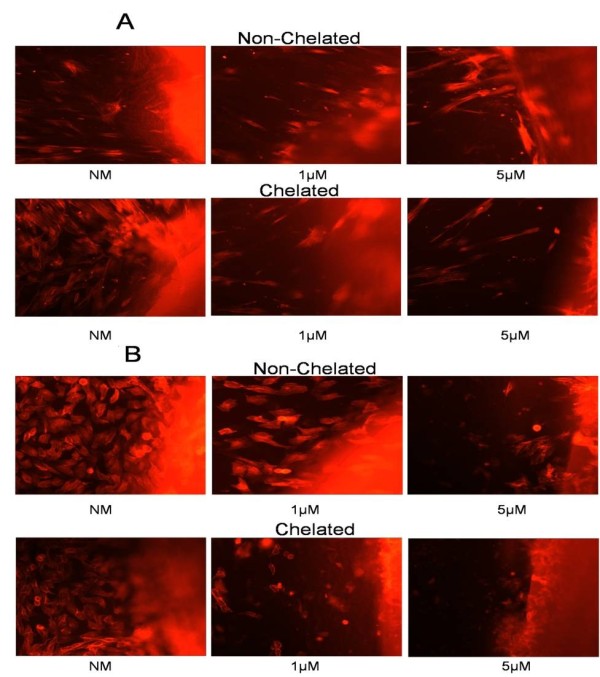
**Microscopical analysis of apoptosis in cells stained with rhodamine**. Fluorescent microscopy images of **(a) **human gingival fibroblast (HGF) and **(b) **HaCaT cells stained with rhodamine, which had been incubated with DDs treated with either normal medium (NM) with infusion solution or ZA 1 or 5 μM (shown as red fluorescence at the right of the image) and then either left unchelated (top rows) or exposed to EDTA 0.001% (chelated) (bottom rows). Images show the aggregation and breakdown of cells, both indicators of apoptosis, after exposure to DDs treated with ZA for 24 h and chelated. Cells were observed over 24 hours with images taken at 24 hours (original magnification ×400).

Physical signs of apoptosis were assayed in individual human gingival fibroblast (HGF) (Figure 1a) and HaCaT (Figure 1b) cells exposed to dentine discs (DDs) treated with NM alone (control) or ZA (0.5, 1, 3 or 5 μM), which either were left unchelated (Figure 1a,b; top rows) or exposed to EDTA 0.001% (chelated) (Figure 1a,b; bottom rows), Results were determined using fluorescent microscopy on rhodamine-stained cells. A negative control for the effects of chelators on cells did not produce any change in apoptotic patterns or in proliferation for either cell line. At 24 hours in the non-chelated model, only a few HGF (5 μM) and HaCaT (3 and 5 μM) cells appeared to aggregate, fragment, and begin showing dead floating cells, indicating apoptosis. By contrast, the chelated model, representing ZA released from bone, showed HGF and HaCaT cells entering apoptosis at a low concentration of ZA (0.5 μM), and there was a marked ring of dead cells visible at 3 and 5 μM (representative concentrations shown).

#### Vital stain to confirm cell death

**Figure 2:**
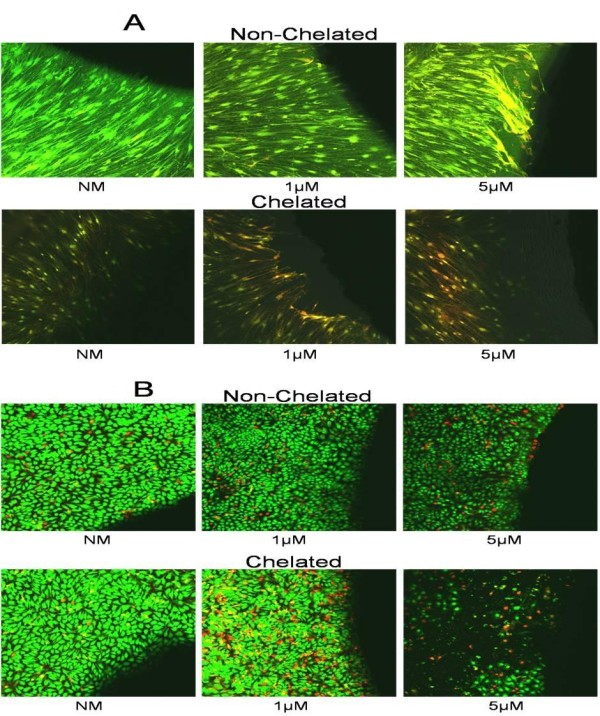
**Vital stain**. Confocal microscopy of **(a) **human gingival fibroblast (HGF) and **(b) **HaCaT cells exposed to DDs treated with either normal medium (NM) with infusion solution or ZA 1 or 5 μM (shown as a lack of fluorescence at the right of the image) and either left unchelated (top rows) or exposed to EDTA 0.001% (chelated) (bottom rows). Images show cells that are viable (green), cells initiating cell death (yellow) and cells that are dying (red) Cells were observed over 24 hours with images taken at 24 hours (original magnification ×400).

To confirm that the physical signs of apoptosis seen using fluorescent rhodamine actually indicated cell death, individual HGF and HaCaT cells exposed to NM (control) or ZA (0.5, 1, 3 or 5 μM) DDs, which either were chelated (EDTA 0.001%) or non-chelated, were examined using vital stain and confocal microscopy. At 24 hours, both HGF (5 μM; Figure 2a, top rows) and HaCaT (3 and 5 μM; Figure 2b, top rows) cultures showed only a few cells initiating cell death (yellow) and dying (red) in the non-chelated model (12 and 25 cells per high power field (HPF), respectively). By contrast, the chelated model showed both HGF (Figure 2a, bottom rows) and HaCaT (Figure 2b, bottom rows) cells entering cell death at ZA 1 μM, with a marked ring of dead cells apparent at ZA 3 and 5 μM (HGF: 168 and 74 cells per HPF; HaCaT: 82 and 93 cells per HPF, respectively) (representative concentrations shown).

### Confirmation of apoptosis

**Figure 3:**
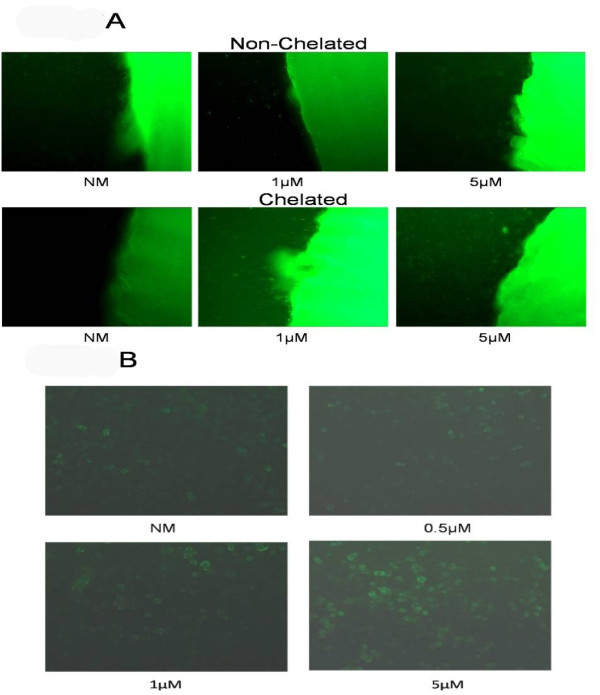
**Apoptosis**. Terminal dUTP nick end labeling assay. **(a) **Fluorescent microscopy images of human gingival fibroblast (HGF) cells exposed to DDs treated with either normal medium (NM) with infusion solution or ZA 1 or 5 μM (green area of fluorescence at the right of the image) and either left unchelated (top rows) or exposed to EDTA 0.001% (chelated) (bottom rows). **(b) **Alternate TUNEL assay was performed in HaCaT cells using first, DDs treated with either normal medium or 0.5, 1 or 5 μM of ZA and exposed to EDTA 0.001% (chelated). Secondly, the medium from the treated DDs was removed and applied to fresh HaCaT cells for 24 hours, followed by TUNEL assay and images using fluorescent microscopy. Cells were observed over 24 hours with images taken at 24 hours. Original magnification (a) ×200; (b) × 400.

**Figure 4:**
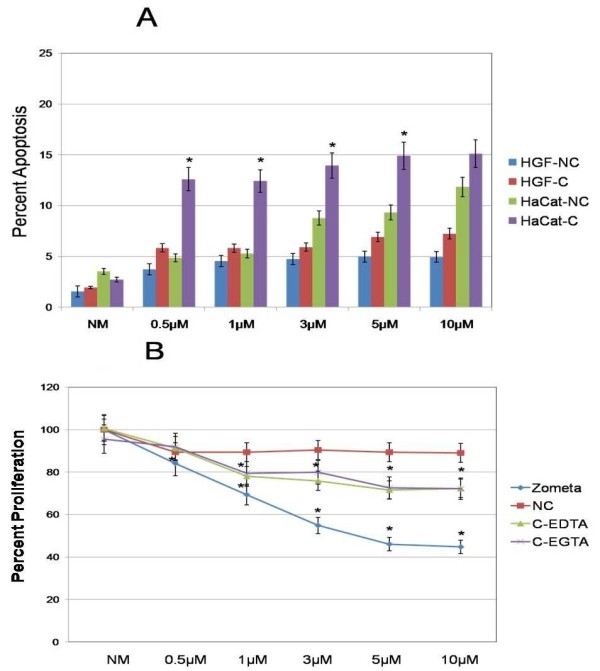
**Annexin V**. **(a) **Annexin V apoptosis assay was performed in human gingival fibroblast (HGF) and HaCaT cells. First DDs treated with either normal medium in infusion solution (NM) or ZA 0.5, 1, 3, 5 or 10 μM and either left unchelated or exposed to EDTA 0.001% (chelated) were assayed. Secondly, the medium from the non-chelated (NC) or chelated (C) DDs was removed and applied to fresh HGF or HaCaT cells for 24 hours. **(b) **Cell proliferation results using mitochondrial tetrazolium salt (MTS) assay. First, DDs treated with either normal medium in infusion solution (NM) or ZA 0.5, 1, 3, 5 or 10 μM and either left unchelated or exposed to EDTA or EGTA 0.001% (chelated) were assayed. Secondly, the medium from the non-chelated (NC) or chelated (C-EDTA or C-EGTA) DDs was removed and applied to fresh HaCaT cells for 24 hours. Error bars indicate SEM. The growth of cells in NM was set as the control. *Significant (*P *< 0.05) differences from control cells.

The dose dependent-increase in apoptosis in the ZA-treated HGF and HaCaT cells observed by microscopy was corroborated by the results from terminal uridine deoxynucleotidyl transferase (dUTP) nick end labeling (Figure 3a,b) and annexin V (Figure 4a) flow cytometry apoptosis assays. Initial confirmation of apoptosis was made using TUNEL assay with HGF and HaCaT cells exposed to DDs treated with NM (control) or ZA (0.5, 1, 3 or 5 μM), which were chelated (EDTA 0.001%) or non-chelated, and viewed by fluorescent microscopy. At 24 hours, at ZA 5 μM, only a few cells were initiating cell death (21 cells per HPF; green) in the non-chelated model (Figure 3a, top rows), whereas in the chelated model, cells started entering cell death at 1 μM (68 cells per HPF), with a marked ring of dead cells apparent at 3 and 5 μM (98 and 147 cells per HPF, respectively) (Figure 3a, bottom rows; shows effects on HGF cells, representative concentrations shown).

Proving proof of principle, a TUNEL assay was performed where DDs only (no cells) treated with either NM (control) or ZA (0.5, 1, 3 or 5 μM) for 24 hours and then chelated (EDTA 0.001%) for 24 hours. Secondly, the medium from the treated DDs was removed and applied to fresh HaCaT cells for 24 hours, then assayed by TUNEL, and viewed by fluorescent microscopy. The HaCaT cells treated with the medium alone from ZA-treated and chelated DDs showed an incrementally increased number of cells entering apoptosis (25, 52, 43 and 182 cells per HPF, respectively) (Figure 3b).

Finally, an annexin V apoptosis assay was performed. First, DDs only (no cells) treated with either NM (control) or ZA (0.5, 1, 3, 5 or 10 μM), and either chelated (EDTA 0.001%) or non-chelated, were assayed. Secondly, the medium from the chelated or non-chelated DDs was removed and applied to fresh HGF or HaCaT cells for 24 hours. Both fresh HGF and HaCaT cells exposed to medium from non-chelated DDs treated with ZA demonstrated an increase in apoptosis (HGF: 2.97%, 3.19%, 3.42% and 3.39% increase for HGF and 1.75%, 5.27%, 5.79% and 8.32% increase for HaCaT at ZA 1, 3, 5 and 10 μM, respectively) compared with the NM control (raw data score of control cultures were averaged over three separate runs: 156 and 352 cells per 10,000 events in HGF and HaCaT cells respectively). Cells exposed to medium from chelated DDs had a larger increase (3.89%, 3.88%, 3.98%, 4.97% and 5.3% increase for HGF and 9.88%, 9.69%, 11.22%, 12.91% and 12.83% for HaCaT at ZA 0.5, 1, 3, 5 and 10 μM, respectively) compared with the NM control (raw data score of control cultures were averaged over three separate runs: 194 and 272 cells per 10,000 events in HGF and HaCaT cells respectively) (Figure 4a). The differences between non-chelated and chelated DDs were significant (*P *< 0.05) for HaCaT cells at all four concentrations of ZA (0.5, 1, 3 and 5 μM).

### Cell proliferation

Cell proliferation using mitochondrial tetrazolium salt (MTS) assay was performed on both cell lines using DDs treated with either NM (control) or ZA (0.5, 1, 3, 5 or 10 μM, and either chelated (EDTA or EGTA 0.001%) or non-chelated. Secondly, the medium from the chelated or non-chelated DDs was removed and applied to fresh HaCaT cells for 24 hours. Results at 24 hours showed that fresh HaCaT cells exposed to medium from non-chelated DDs treated with ZA (0.5, 1, 3, 5 and 10 μM) failed to demonstrate a significant difference in cell proliferation from the NM control (raw data score of control cultures were averaged over three separate runs: 156 and 352 cells per 10,000 events in HGF and HaCaT cells respectively)(Figure 4b). The chelated models of fresh HaCaT cells exposed for 24 hours to medium from chelated DDs demonstrated a combined decrease in cell proliferation (12%, 14%, 19.4%, 19.9% decrease at ZA 1, 3, 5 and 10 μM, respectively) compared with the NM control (raw data score of control cultures were averaged over three separate runs: 156 and 352 cells per 10,000 events in HGF and HaCaT cells respectively) (Figure 4b). The level of ZA released from the DDs was determined by comparing levels of ZA in the medium with the effects on cell proliferation induced by direct addition of ZA from our previous studies [14,28,32]. We found that levels of ZA released from DDs secondary to chelation with EDTA/EGTA to be equivalent to 0, 0.25, .075, 0.87, 0.96 and 0.98 μM on average at ZA concentrations of 0, 0.5, 1, 3, 5 and 10 μM, respectively, for both chelation methods. Significant differences from controls were seen in the chelated DD model starting at the 1 μM level, whereas cells treated directly with ZA were significantly different from controls at the 0.5 μM level (*P *< 0.05). The non-chelated DDs never demonstrated significant differences from NM controls.

## Discussion

Currently, over 190 million prescriptions oral BP are dispensed worldwide, with an increasing incidence of reported cases of ONJ [33]. Most reported cases of ONJ (94%) are in patients receiving intravenous BPs for supportive cancer care [18], particularly patients with multiple myeloma, metastatic prostate cancer and metastatic breast cancer. Recently, however, cases of ONJ were also reported in patients treated with oral alendronate for osteoporosis [18]. The use of the IV formulation of ZA every 3 to 4 weeks appears to be associated with the greatest risk for developing ONJ [18,30]. However, the exact mechanism by which this occurs is not yet known [34]. It is hypothesized that the skeleton acts as a reservoir of BP that produces high concentrations located within the vicinity of the bone [27]. Interestingly, treatment of osteoclasts, monocytes and tumor cells with BP in solution leads to cell death, indicating that resorption of bone is not required for cellular BP uptake. Osteoclasts treated with BP are shown to lose function to the same degree as those exposed to BP-treated bone slices, indicating that bone resorption is not necessary for BP action [35].

In this study, we developed an *in vitro *model showing for the first time proof of and the mechanism for a soft-tissue component of ONJ, whereby after release from bone, BP induces gingival fibroblast and epithelial cell death via apoptosis, with the continued release of ZA preventing spontaneous resolution or healing, due to inhibition of cell proliferation. Recognizing various potential chelating interactions with the surrounding mucosal and osseous tissues, we believe this process results in increased mucosal cell apoptosis, leading to eventual thinning and exposure of bone, allowing for rapid microbial colonization [36]. The formation of a biofilm smear layer would create an environment of mild to moderate osteomyelitis, preventing the resolution of inflammation and healing in the oral cavity, therefore leading to persistent ONJ [36]. Additionally, calcium chelators in saliva may produce a localized release of BP, further inducing cell apoptosis and providing a mechanism that would explain the prolonged length of bone exposure and failure of healing [32,37]. Microscopy, TUNEL, vital stain and annexin V studies demonstrated that ZA chelated from DDs leads to dose-dependent apoptotic processes in human epithelial (HaCaT) and fibroblast (HGF) cells. These data point to a direct effect of ZA on epithelium and fibroblasts in a temporal sequence that begins at very low concentrations (1 μm) of BP in the fibroblasts, and then proceeds to the epithelial cells.

Interestingly, in this model, proliferation studies demonstrated a reduced amount of cell growth inhibition with up to 10 μM ZA, in contrast to previous studies, which showed significant inhibition at 1-5 μM [28,32,37]. A possible explanation for this discrepancy is the amount of ZA released upon chelation from the DDs. Currently, a mechanism to detect the level of ZA in medium, saliva, bone or blood does not exist. Our model uses a mechanism to determine the maximum dose of ZA administered, assuming that the level may be lower than that applied to the DDs. However, with this in mind we have shown that levels potentially lower than those previously shown can induce changes in the oral mucosa that are sufficient to produce mucosal dehiscence and ONJ lesions [28,32]. Confirming this, the annexin V assays using medium from chelated ZA-treated DDs produced significant apoptosis in fresh mucosal cells not exposed to DDs.

Our *in vitro *model supports our hypothesis that ONJ occurs via a combined mechanism, including bone, systemic and soft-tissue mechanisms. The bony mechanism is believed to occur secondary to an alteration of angiogenesis of new osseous tissue, inducing detrimental effects on the quality and quantity of bone perfusion, and ultimately leading to an altered response of osseous tissue to trauma, infection and wound healing [35]. Incorporation of the systemic alterations caused by the additional pharmacologic therapies that this type of patient must undergo, would prevent exposed bone from fighting infections in the oral cavity, perpetuating the risk of ONJ [38]. Finally, the release of BP from bone secondary to calcium chelation could initiate the process via mucosal dehiscence.

Further studies are needed to fully elucidate this model, including the biochemical mechanisms, the effect on dual cell lines of epithelial and fibroblast origin and the effect of induced osteoclasts, and to determine if such effects are clinically relevant for *in vivo *models of ONJ.

## Conclusions

The importance of this study rests on the fact that only empirical treatment is currently employed for ONJ. Currently, preventive strategies aimed at avoiding invasive oral interventions such as dental surgery and subsequent infection are the standard of care. Additionally, it is recommended that until healing from an invasive dental surgical treatment takes place, temporary discontinuation of BP therapy (up to 3 months) may be considered [39,40]; however there is no prospective evidence that this affects outcome. These weakly supported treatment recommendations flow from a clinical paradigm and fail to follow a causative mechanism. We provide for the first time an *in vitro *model showing that the direct contact of clinically relevant concentrations of ZA with epithelial or fibroblast cells induces apoptosis and prevents cell proliferation, with the potential result being ONJ. It is our expectation that expanding this *in vitro *model, including oral microbes, will produce a model mimicking the oral environment, so as to conceive and design specific interventions, and produce *in vivo *studies to determine oral ZA levels in crevicular and salivary fluids, which will further confirm this hypothesis.

## Methods

### Cell lines and cell cultures

All experiments were performed using an established human keratinocyte cell line (HaCaT) and a human gingival fibroblast cell line (HGF) (donated by Dr Silvio Gutkind at the National Institutes of Health and John Sauk at University of Maryland, respectively), selected as cells representative of the unique oral mucosa. Other researchers have observed the effects of BP on murine oral keratinocytes, cancer cells and osteoclasts, showing cell line apoptosis and/or decreased cell proliferation [41-46]; however, these studies were not specific to the disease process, and because ONJ is a specific disease state, we chose specific native cell lines for our model. Cells were cultured in (i) Dulbecco modified Eagle medium (DMEM) with 10% fetal bovine serum (FBS), penicillin 100 U and streptomycin 100 μg/ml or (ii) a 1:1 mix of Ham F12 and DMEM with 10% FBS, penicillin 100 U, streptomycin 100 μg/ml and hydrocortisone1.0 mg/ml (Sigma Chemical Company, St. Louis, MO, USA), respectively. The cells were cultured at 37°C in a 5% CO_2 _in air atmosphere until confluent, and subcultured using a disaggregation assay with trypsin 0.1% and EDTA 0.01% in PBS (pH 7.5). For all experiments, cells were grown in 6-, 24-, 48- or 96-well plates at 5 × 10^4 ^cells per well and grown to 80% to 90% confluence. Control cells (in NM) were treated for all experiments with the infusion solution (0.36% saline, which did not contain calcium) alone in NM, in accordance with the manufacturer's directions. Saline was used to closely mimic the true infused drug used in the clinical setting. All experiments were performed in triplicate, and repeated on two separate occasions.

### Drug treatments

Injectable ZA (Zometa; Novartis Pharmaceuticals Corp, East Hanover, NJ, US) was selected for these studies because it is the most widely reported BP associated with ONJ. The model established here is applicable to further investigations with all types of BP. ZA was used for all experiments at six different concentrations (0, 0.5, 1, 3, 5, 10 μM), These concentrations were selected because they are clinically relevant to patients receiving ZA, being representative of the lower limits of estimated plasma concentrations after a 15-minute infusion (baseline plasma concentration level is 1 μM). First, ZA was diluted to the appropriate concentration in NM. For each drug concentration, one DD (our mimic of bone) (IDS Ltd., Bolden, Tyne and Wear, UK), was used. The DDs were immersed in the drug in 48-well plates for 24 hours. They were then removed from the normal or ZA-containing medium, washed three times in PBS and inserted into 6- or 24-well plates containing fresh medium without ZA added. Next, HaCaT or HGF cells were plated into the same wells as the DDs and allowed to grow onto and around the DDs to 90% confluence. The ZA (0.5 to 10 μM) treated and untreated (ZA 0 μM) DDs were then either left unchelated or were chelated using EDTA or EGTA at 0.001%. The chelating agents were selected for their known activity and their simulation of the oral environment, as various components of saliva are capable of chelation. EDTA and EGTA were selected to validate our model, as the additional components of saliva might confound our *in vitro *model. Cells were visualized, photographed and assayed during the 24 hour treatment, with images collected showing the zone of apoptosis surrounding the DDs. Controls for the use of EDTA and EGTA were DDs pretreated in NM alone without ZA, which showed minimal apoptosis.

### Direct microscopical observation

Adherent HaCaT and HGF cell lines were plated in fresh medium (no ZA) overlying and surrounding either untreated (NM) or treated DDs (ZA 0.5, 1, 3 or 5 μM concentration diluted in infusion solution), which were then either left unchelated or were chelated using EDTA 0.001% for 24 hours. Cells were examined under a microscope (Axiovert 135; Zeiss, Thornwood, NY, USA) hourly for up to 24 hours (images not shown).

### Rhodamine assay

Adherent HaCaT and HGF cell lines were plated into fresh medium (no ZA) overlying and surrounding either untreated (NM) or treated DDs (0.5, 1, 3 or 5 μM ZA diluted in infusion solution), which were then either left unchelated or were chelated using EDTA 0.001% for 24 hours. After 24 hours, cells were washed twice with PBS and fixed with a solution of 4% paraformaldehyde in PBS pH 7.4 for 1 h at 20°C. Cells were rinsed twice with PBS and then incubated with permeabilisation solution for 2 minutes on ice. The cells were rinsed in PBS and labeled with rhodamine for 30 minutes, rinsed twice with PBS, and examined under a fluorescent microscope with a detection range of 515 to 565 nm.

### TUNEL staining

Adherent HaCaT and HGF cell lines were plated into fresh medium (no ZA) overlying and surrounding either untreated (NM) or treated DDs (0.5, 1, 3 or 5 μM ZA diluted in infusion solution), which were then either left unchelated or were chelated using EDTA 0.001% for 24 hours. Cells were washed twice with PBS and fixed with a fixation solution of paraformaldehyde 4% in PBS pH 7.4 for 1 h at 20°C. Cells were rinsed twice with PBS and then incubated with permeabilisation solution for 2 minutes on ice. The cells were rinsed in PBS and labeled using 50 μl of a 9:1 solution of 'Label and Enzyme solution' (In Situ Cell Death Detection Kit, Fluorescein; Roche Applied Sciences, Mannheim, Germany), with appropriate controls labeled only with the Label solution. The cells were incubated for 1 h at 37°C in a humidified atmosphere in the dark, rinsed in PBS, and examined under a fluorescent microscope with a detection range of 515 to 565 nm.

### Vital stain

Adherent HaCaT and HGF cell lines were plated in fresh medium (no ZA) overlying and surrounding either untreated (NM) or treated DDs (0.5, 1, 3 or 5 μM ZA diluted in infusion solution), which were then either unchelated or were chelated using EDTA 0.001%.for 24 hours. Cells were washed twice with PBS and stained to evaluate cell membrane integrity (BacLight Vital Bacterial Viability Kit; cat. no. L-7012; Molecular Probes, Eugene, OR, USA). The kit contains Syto9 to detect cells with intact membranes (live) and propidium iodide to identify membrane-damaged cells (dead) [47] The stain was prepared by dilution of 3 μl of each component into 1 ml of Tris-EDTA buffer (pH 7.0). Confocal microscopy was used to analyze the rate of cell death.

### Flow cytometry and annexin V studies

Apoptosis was evaluated using annexin V conjugated with fluorescein isothiocyanate. DDs alone were placed in 48-well plates with either untreated (NM) or treated (ZA of 0.5, 1, 3, 5 or 10 μM concentration diluted in fresh medium with infusion solution) medium for 24 h. After 24 h, the DDs were removed, washed three times in PBS and placed in new 6-well plates with fresh medium (no ZA) and either were left unchelated or were chelated using EDTA 0.001% for 24 hours. In separate 6-well plates (no DD), HaCaT and HGF cells were grown to 80% confluency. Cells were washed with Hank's balanced salt solution (HBSS) and then treated using the medium only from the initially untreated or ZA-treated and non-chelated or chelated DD wells. Next, the cells were washed twice with PBS and washed with HBSS, followed by lysis using 0.1% trypsin and 0.01% EDTA in PBS pH 7.5. The cells were washed with cold PBS and resuspended in 1× binding buffer (BD-Pharmingen Biosciences, San Diego, CA, USA), then 5 μl of annexin and 5 ml of propidium iodide were added to the cells, which were then mixed by vortex and incubated for 15 minutes in the dark. Finally, 400 ml of 1× binding buffer was added, and samples were evaluated by flow cytometry (Epics Elite ESP Flow Cytometer; Beckman Coulter, Brea, CA, USA).

### Cell proliferation

Proliferation was performed by non-radioactive MTS cell proliferation assay (Promega, Madison, WI, USA). DDs alone were placed in 48-well plates and either left untreated (NM) or treated with ZA (0.5, 1, 3, 5 or 10 μM concentration diluted in fresh medium with infusion solution) for 24 hours. The DDs were then removed, washed three times in PBS and placed in new 6-well plates with fresh medium (no ZA) and either were left unchelated or were chelated using EDTA or EGTA 0.001% for 24 hours. HaCaT cells were separately plated by themselves in 96-well plates using a density of 2,500 cells/well and allowed to grow to 80% confluence. Cells were washed with HBSS and then treated using the medium only from the initially untreated or ZA treated and then non-chelated or chelated DDs. The cells were then examined by MTS cell proliferation assay, which was read at a wavelength of 570 nm (96-well MR4000 Microplate Palate Reader; Dynatech, Chantilly, VA, USA). The percentage cell growth was determined by setting as 100% the growth of control cells treated only with infusion solution in NM. All analyses were performed in triplicate and repeated on separate occasions.

### Statistical analysis

For all measurements, as needed, *t*-test or Student's *t*-test or was used to assess significance of treated groups versus control groups. *P *< 0.05 was considered significant, and data are presented as means ± SEM.

## Competing interests

The authors declare that they have no competing interests.

## Authors' contributions

MS carried out molecular studies, performed microscopic imaging, drafted the manuscript and oversaw all aspects of the project. RC carried out molecular studies including PCR arrays and MTS assays. KC contributed to the discussion of the potential clinical significance of this project and assisted in editing the manuscript. These projects grew out of discussions between TM and MS. TM participated in the design and coordination of all projects and final editing of the manuscript. All authors read and approved the final manuscript.
